# Changes in Patient Safety Knowledge During Undergraduate Nursing Education: A Scoping Review Protocol

**DOI:** 10.3390/nursrep14040266

**Published:** 2024-11-22

**Authors:** João Daniel de Souza Menezes, Matheus Querino da Silva, Yuri Sacardo, Rodrigo Soares Ribeiro, Stela Regina Pedroso Vilela Torres de Carvalho, Emerson Roberto dos Santos, Marcos Sanches Rodrigues, André Bavaresco Gonçalves Cristóvão, Nathalia Bavaresco Gonçalves Cristóvão, Helena Landim Gonçalves Cristóvão, Alexandre Lins Werneck, Alex Bertolazzo Quitério, Ana Caroline Santos Costa, Emilia Batista Mourão Tiol, Isabela Amaral Almeida Bistafa, Sônia Maria Maciel Lopes, Marli Carvalho Jerico, Patrícia da Silva Fucuta, Marielza Regina Ismael Martins, Clemente Neves Sousa, Tiago Filipe Cardoso Oliveira Casaleiro, Vânia Maria Sabadoto Brienze, Alba Regina de Abreu Lima, Rita Cássia Helú Mendonça Ribeiro, Júlio César Andre

**Affiliations:** 1 Center for Studies and Development of Health Education, Faculty of Medicine of São José do Rio Preto (CEDES/FAMERP), Av. Brigadeiro Faria Lima, 5416—Vila Sao Pedro, São José do Rio Preto 15090-000, Brazil; matheusquirino@hotmail.com (M.Q.d.S.); stelarpvs@hotmail.com (S.R.P.V.T.d.C.); emerson.santos@edu.famerp.br (E.R.d.S.); fisiosanches@yahoo.com.br (M.S.R.); alex.quiterio@edu.famerp.br (A.B.Q.); ana.scosta@edu.famerp.br (A.C.S.C.); emilia.tiol@edu.famerp.br (E.B.M.T.); isabela.bistafa@edu.famerp.br (I.A.A.B.); sonia.lopes@edu.famerp.br (S.M.M.L.); patricia@fukuta.com.br (P.d.S.F.); clementesousa@esenf.pt (C.N.S.); tcasaleiro@esesfm.pt (T.F.C.O.C.); vania.brienze@hospitaldebase.com.br (V.M.S.B.); alba.lima09@gmail.com (A.R.d.A.L.); julio.andre@edu.famerp.br (J.C.A.); 2 Postgraduate Department, Faculty of Medicine of São José do Rio Preto, Av. Brg. Faria Lima, 5416—Vila Sao Pedro, São José do Rio Preto 15090-000, Brazil; enfyurisacardo@gmail.com (Y.S.); merodrigoribi@gmail.com (R.S.R.); alexandrelinswerneck@gmail.com (A.L.W.); marli@famerp.br (M.C.J.); marielzamartins@famerp.br (M.R.I.M.); ritadecassia@famerp.br (R.C.H.M.R.); 3 Medicine Department, University of Santo Amaro, Rua Isabel Schmidt, 349-Santo Amaro, São Paulo 29060-120, Brazil; andre.cristovao017@gmail.com (A.B.G.C.); nathaliacristovao10@gmail.com (N.B.G.C.); helenalgc@gmail.com (H.L.G.C.); 4 Medicine Department, Faceres Medical School, Av. Anísio Haddad, 6751—Jardim Francisco Fernandes, São José do Rio Preto 15090-305, Brazil

**Keywords:** patient safety, nursing education, undergraduate nursing students, knowledge acquisition, curriculum development, scoping review protocol

## Abstract

Background/Objectives: Patient safety is a critical component of healthcare quality, yet there remains a significant gap in understanding how patient safety knowledge progresses among nursing students throughout their educational journey. This scoping review aims to map and analyze the existing literature on the development of patient safety knowledge in undergraduate nursing education. Methods: This study will follow the Joanna Briggs Institute methodology for scoping reviews and adhere to the PRISMA-ScR (Preferred Reporting Items for Systematic Reviews and Meta-Analyses—Scoping Reviews) guidelines. A comprehensive search strategy will be employed across multiple databases, including CINAHL (Cumulative Index to Nursing and Allied Health Literature), ERIC (Education Resources Information Center), PubMed, Embase, SCOPUS, and Web of Science. The review will include studies published from 2019 to 2024 in English, Portuguese, and Spanish. Two independent reviewers will conduct study selection and data extraction. The data will be synthesized narratively, with quantitative data summarized using descriptive statistics and qualitative data analyzed thematically. Results: The review is expected to identify key patterns in the progression of patient safety knowledge among nursing students, including critical periods for knowledge acquisition, challenges in translating theoretical knowledge into practice, and effective educational strategies. The findings will be presented in both tabular and narrative forms, providing a comprehensive overview of the current state of patient safety education in nursing programs. Conclusions: The anticipated outcomes of this review have significant implications for nursing education, healthcare policy, and clinical practice. For educators, the findings will inform curriculum development and the design of targeted interventions to enhance patient safety competencies. From a policy perspective, the results could support the standardization of patient safety education across nursing programs. In clinical practice, the study may emphasize the importance of continuous professional development in patient safety. This scoping review aims to fill a critical gap in the literature by providing a comprehensive understanding of how patient safety knowledge progresses among nursing students. The findings are expected to contribute significantly to the advancement of nursing education and patient safety, ultimately fostering a culture of safety that benefits both healthcare providers and recipients.

## 1. Introduction

Patient safety is a fundamental component of healthcare quality, aiming to reduce health-related risks and avoidable harm to an acceptable minimum [1]. The topic gained prominence with the publication of the Institute of Medicine’s “To Err is Human” report and the creation of the World Alliance for Patient Safety by the World Health Organization in 2004 [2,3].

Recent estimates indicate that medical errors, including those related to nursing care, cause significant harm and mortality worldwide. A systematic review found that the pooled prevalence of preventable patient harm was 6%, with a pooled proportion of preventable deaths of 0.23% [4]. In low- and middle-income countries, the burden of patient harm is even higher, with up to 8% of hospitalizations resulting in adverse events [5].

These alarming data reinforce the need for solid patient safety training for healthcare professionals, especially nurses, who play a central role in preventing and mitigating errors and harm to patients [6]. Studies have shown that providing patient safety education to nurses can lead to improvements in safety culture, reduced adverse events, and better patient outcomes [7,8].

The inclusion of patient safety content in nursing curricula can significantly improve nursing students’ knowledge, skills, and attitudes regarding this theme [9,10]. This scoping review focuses specifically on changes in knowledge, as knowledge is a fundamental precursor to developing appropriate skills and attitudes related to patient safety [11].

Despite the recognized importance of patient safety in healthcare education, there remains a critical gap in our understanding of how patient safety knowledge progresses among nursing students throughout their educational journey. This scoping review aims to address this gap by carrying out the following steps:

1. Systematically mapping the existing literature on patient safety knowledge acquisition in nursing education;
2. Identifying key stages and factors influencing the progression of patient safety knowledge;
3. Exploring potential discrepancies between theoretical knowledge and practical application in clinical settings;
4. Evaluating the effectiveness of current educational strategies in fostering patient safety competencies.

Understanding this progression is vital for the following reasons:

(a) It can inform curriculum development, ensuring that patient safety education is appropriately scaffolded throughout nursing programs;
(b) It may highlight critical periods for interventions in or the reinforcement of patient safety concepts;
(c) It can guide the development of more effective teaching strategies and assessment methods;
(d) It has the potential to impact healthcare policy by providing evidence for standardization of patient safety education in nursing curricula.

Recent studies have highlighted the need for a more nuanced understanding of patient safety knowledge progression, particularly in light of evolving healthcare technologies and practices. For instance, Lee and Dahinten (2023) [12] emphasized the importance of integrating patient safety concepts throughout the nursing curriculum, while Bartoníčková et al. (2024) [13] identified gaps in how patient safety knowledge is translated into clinical practice by nursing students. This review seeks to synthesize current evidence and identify areas for future research, ultimately contributing to improved patient safety outcomes through enhanced nursing education.

## 2. Justification

A preliminary search in CINAHL and MEDLINE databases found a recent systematic review on educational interventions to develop patient safety competencies in nursing students [14]. However, the review focused on the effectiveness of specific interventions rather than mapping the broader literature on changes in knowledge throughout the undergraduate trajectory. The present scoping review will fill this gap by providing a more comprehensive picture of the current state of evidence.

The results of this review may provide a solid evidence base to inform educators, researchers, and policymakers about promising practices in teaching patient safety to nursing students, as well as identify gaps in current knowledge and promising areas for future research.

## 3. Materials and Methods

This scoping review will be conducted in accordance with the Joanna Briggs Institute (JBI) methodology for scoping reviews [15]. The review process and reporting will follow the Preferred Reporting Items for Systematic Reviews and Meta-Analyses extension for Scoping Reviews (PRISMA-ScR) checklist [16].

The implementation of these guidelines will encompass the following methodological steps:

1. The development of a comprehensive protocol prior to initiating the review, as recommended by JBI;
2. The formulation of a clear research question utilizing the PCC (Population, Concept, and Context) framework;
3. The explicit delineation of inclusion and exclusion criteria aligned with the research question;
4. The construction of an exhaustive search strategy in collaboration with a specialized research librarian;
5. The execution of a systematic search across multiple databases, including gray literature sources;
6. A two-stage study selection process (title/abstract screening followed by full-text review) conducted independently by two reviewers;
7. Data extraction using a standardized form specifically designed for this study;
8. The critical appraisal of the included studies, although this is not a mandatory step in scoping reviews, to provide additional contextual information;
9. The narrative synthesis of results, incorporating tables and figures for clear data presentation;
10. Transparent reporting adhering to the PRISMA-ScR checklist.

This methodological approach ensured a comprehensive, systematic, and transparent review process, aligning with the highest standards of scoping review methodology.

### 3.1. Review Objective and Question

The objective of this scoping review is to identify, map, and present the information available in the literature about changes that occur during undergraduate nursing education regarding knowledge about patient safety.

The review question is: “What is known from the existing literature about changes in undergraduate nursing students’ knowledge about patient safety during their education?”

- Change in knowledge about patient safety during undergraduate nursing education. Knowledge will be assessed using Kirkpatrick’s evaluation model, focusing on Level 2 (learning outcomes) [17].
- Context: Any undergraduate nursing educational setting.
- Types of sources: Original articles, theoretical studies, experience reports, clinical study articles, case studies, normative, integrative, and systematic reviews, meta-analyses, and meta-syntheses in English, Portuguese, and Spanish from 2019 to 2024. Gray literature sources will be searched in appropriate databases.

### 3.2. Concept

The concept of interest is knowledge and attitudes about patient safety, attributes that healthcare professionals must develop to act preventively and promote a positive safety culture. Results assessing the levels of these aspects will be included, and those without mention of the topic will be excluded.

### 3.3. Search Strategy

The search strategy was carefully designed to balance comprehensiveness and specificity, ensuring that relevant literature will be captured without overwhelming the review with extraneous results. Boolean operators were chosen based on preliminary searches and expert consultation, allowing for flexibility across different databases. Search terms were selected through an iterative process, incorporating both controlled vocabulary and free-text terms to maximize retrieval. This strategy was validated through pilot searches in each database, confirming its effectiveness in retrieving pertinent studies in the future.

The databases to be searched include CINAHL (EBSCO), ERIC (EBSCO), PubMed, Embase, SCOPUS, and Web of Science. The search strategy will include terms related to nursing students, patient safety, and knowledge. The complete search strategies for each database are provided in Table 1.

### 3.4. Study Selection

After the search, all identified records will be collated and uploaded into the Rayyan systematic review software [18], and duplicates will be removed. Titles and abstracts will then be screened by two independent reviewers. Potentially relevant studies will be retrieved in full, and their citation details imported into Rayyan. The study selection process will be conducted by two independent reviewers (J.D.M. and M.Q.S.). These reviewers will perform the initial screening of titles and abstracts, as well as the full-text review of potentially relevant articles. In cases of disagreement between the two primary reviewers, a third reviewer (Y.S.) will be consulted to arbitrate and reach a final decision. This rigorous approach will ensure an impartial assessment of the evidence, minimizing potential selection bias. The involvement of multiple reviewers will enhance the reliability and validity of the study selection process, adhering to best practices in systematic review methodology.

### 3.5. Data Extraction

The data extraction process will involve collecting specific information on study characteristics, participant demographics, intervention types, and outcomes measured (Table 2). Data will be categorized into quantitative and qualitative types. Quantitative data will be analyzed using descriptive statistics, including means, medians, and standard deviations, to summarize key findings across studies. Qualitative data will be synthesized thematically to identify common patterns and themes. Discrepancies between studies will be addressed through sensitivity analyses and subgroup analyses, where appropriate, to assess the robustness of the findings.

### 3.6. Quality Appraisal

While a formal quality appraisal is not usually conducted in scoping reviews, we will provide a general assessment of the strength of evidence for our key findings, based on the study design and methodological limitations.

## 4. Presentation of Results

Results will be presented in both tabular and narrative forms. Tables will provide a summary of the extracted data, highlighting key variables and outcomes across studies. Narrative synthesis will offer a detailed interpretation of the results, integrating quantitative and qualitative findings to provide a comprehensive overview of the research landscape.

## 5. Discussion

This scoping review aims to comprehensively map and analyze the progression of patient safety knowledge among nursing students throughout their educational journey. By synthesizing the existing literature, this study seeks to provide valuable insights into the development of patient safety competencies in nursing education.

### 5.1. Summary of Findings

While the specific results of this review are yet to be determined, we anticipate identifying key patterns in how patient safety knowledge evolves during nursing education. These patterns may include critical periods for knowledge acquisition, common challenges in translating theoretical knowledge into practice, and effective educational strategies that enhance patient safety competencies.

### 5.2. Comparison with Existing Literature

Our findings are expected to build upon existing research in patient safety education. Previous studies, such as those by Lee and Dahinten (2023) [12] and Bartoníčková et al. (2024) [13], have highlighted the importance of integrating patient safety concepts throughout nursing curricula. This review aims to provide a more comprehensive understanding of how this integration occurs and its effectiveness across different educational contexts.

### 5.3. Strengths and Limitations

The strength of this review lies in its comprehensive approach, encompassing a wide range of literature to provide a holistic view of patient safety education in nursing. However, limitations may include the potential for publication bias and the challenge of synthesizing diverse educational approaches across different healthcare systems and cultural contexts.

### 5.4. Implications of the Study

The implications of this study are multifaceted, encompassing both educational and practical dimensions. Educationally, the findings are expected to inform curriculum development by highlighting critical stages in nursing education where patient safety knowledge can be most effectively integrated and reinforced. This can lead to the creation of targeted educational interventions that ensure nursing students acquire comprehensive patient safety competencies throughout their training.

Practically, the study’s results could influence healthcare policy by providing evidence for standardizing patient safety education across nursing programs. By demonstrating the progression of patient safety knowledge, this research can support the development of guidelines that ensure all nursing graduates possess the necessary skills to maintain high standards of patient care. Additionally, understanding these educational trajectories can help identify gaps in current training programs, prompting revisions that address these deficiencies.

Furthermore, the study may have implications for clinical practice by emphasizing the importance of continuous professional development in patient safety. Healthcare institutions could use these findings to enhance their training programs, ensuring that both new graduates and experienced nurses remain up-to-date with best practices in patient safety.

### 5.5. Future Research Directions

This review is likely to identify areas where further research is needed. These may include longitudinal studies tracking patient safety knowledge retention post-graduation, investigations into the effectiveness of specific educational interventions, and exploration of how emerging healthcare technologies impact patient safety education.

## 6. Conclusions

In conclusion, this scoping review aims to elucidate the progression of patient safety knowledge among nursing students, filling a critical gap in the current literature. Beyond merely summarizing the study’s objectives, the anticipated outcomes are poised to offer significant benefits for both nursing educators and healthcare policymakers. By systematically mapping existing educational practices and identifying key stages for effective knowledge integration, this study will provide a framework for developing targeted curriculum enhancements that ensure comprehensive patient safety education.

For nursing educators, the insights gained from this review are expected to inform the design of innovative teaching strategies and assessment methods that align with best practices in patient safety. These strategies will be crucial for equipping future nurses with the competencies necessary to navigate complex clinical environments safely and effectively.

From a policy perspective, the study’s findings could advocate for the standardization of patient safety education across nursing curricula, thereby promoting consistency in training and ultimately improving patient outcomes. The evidence generated could serve as a foundation for policy development, guiding institutions in implementing robust educational standards that prioritize patient safety.

Overall, the completion of this study promises to contribute meaningfully to the advancement of nursing education and patient safety, fostering a culture of safety that benefits both healthcare providers and recipients. This forward-looking approach underscores the study’s potential impact, positioning it as a pivotal resource for driving improvements in educational practices and healthcare policy.

## Figures and Tables

**Table 1:** Search strategies.

Database	Search Strategy
PUBMED	(“patient *” [Title/Abstract] AND “english” [Language] AND ((((“harm *” [Title] NOT “harmon *” [Title]) AND “english” [Language]) OR (“safe *” [Title] AND “english” [Language]) OR (“accident *” [Title] AND “english” [Language])) AND “english” [Language]) AND (“nurs *” [Title] AND “english” [Language]) AND (“know *” [Title/Abstract] AND “english” [Language])) AND (English [Filter])
EMBASE	(patient: ab OR patient: kw OR patient: ti OR patients: ab OR patients: kw OR patients: ti) AND (harm: ti OR harms: ti OR safe *: ti OR accident: ti OR accidents: ti) AND nurs *:ti AND know*: ab
SCOPUS	(TITLE-ABS-KEY (patient *)) AND ((TITLE (safe *)) OR (TITLE (accident *)) OR (TITLE (harm * AND NOT harmon *))) AND (TITLE (nurs *)) AND (TITLE-ABS-KEY (know *))
WEB OF SCIENCE	((TI = (patient *)) OR AB = (patient *)) AND (((TI = (harm *)) NOT TI = (harmon *)) OR TI = (safe*) OR TI = (accident *)) AND TI = (nurs *) AND ((TI = (know *)) OR AB = (know *))
CINAHL (EBSCO)	(TI patient OR AB patient OR TI patients OR AB patients) AND ((TI harm * NOT TI harmon *) OR (TI safe *) OR (TI accident *)) AND (TI nurs *) AND (AB know * OR TI know *)
ERIC (EBSCO)	(TI patient OR AB patient OR TI patients OR AB patients) AND ((TI harm * NOT TI harmon *) OR (TI safe *) OR (TI accident *)) AND (TI nurs *) AND (AB know * OR TI know *)

Source: Author.

**Table 2:** Draft data extraction tool.

Draft Data Extraction Tool
**Study Characteristics:**
1. Author(s);
2. Year of publication;
3. Country of origin;
4. Study design;
5. Study setting;
6. Funding source.
**Population:**
7. Sample size;
8. Age range;
9. Gender distribution;
10. Year of study;
11. Inclusion/exclusion criteria.
**Concept:**
12. Educational interventions or exposures related to patient safety;
13. Duration and frequency of interventions;
14. Delivery methods (e.g., lectures, simulations, online modules);
15. Content areas covered.
**Context:**
16. Undergraduate nursing program characteristics;
17. Curricular integration of patient safety content;
18. Institutional support for patient safety education.
**Methods:**
19. Data collection methods;
20. Outcome measures;
21. Validity and reliability of measurement tools;
22. Data analysis techniques.
**Key Findings:**
23. Changes in patient safety knowledge scores;
24. Factors associated with knowledge acquisition;
25. Retention of knowledge over time;
26. Impact on skills, attitudes, or behaviors related to patient safety;
27. Barriers and facilitators to effective patient safety education.
**Other:**
28. Limitations;
29. Recommendations for future research;
30. Implications for nursing education and practice.

The table “Draft Data Extraction Tool” provides a detailed structure for extracting data from studies on educational interventions related to patient safety in nursing programs. It is organized into several sections, each addressing different aspects of the studies. Source: Author.

## Data Availability

No new data were created or analyzed in this study. Data sharing is not applicable to this article.

## Public Involvement Statement

There was no public involvement in any aspect of this research.

## Guidelines and Standards Statement

This manuscript was drafted considering the PRISMA-ScR guidelines.
